# The addition of collagenase to BromAc^**®**^ for the management of inoperable pseudomyxoma peritonei – *in vitro* results

**DOI:** 10.1515/pp-2025-0026

**Published:** 2025-10-31

**Authors:** Carissa Vici, Mathew Eapen, Pooja Narang, David L. Morris

**Affiliations:** 2989 Peritonectomy Unit, St George Hospital, South Eastern Sydney Local Health District, Kogarah, NSW, Australia; University of New South Wales Saint George and Sutherland Clinical Campuses, Kogarah, NSW, Australia; Mucpharm Pty. Ltd., Kogarah, NSW, Australia

**Keywords:** pseudomyxoma peritonei, collagenase, BromAc^®^, mucin

## Abstract

**Objectives:**

Pseudomyxoma peritonei (PMP) is a rare peritoneal malignancy. BromAc^**®**^ is a novel therapeutic agent which has been developed to dissolve and facilitate the drainage of mucin as a palliative treatment for PMP; however, its effect is significantly reduced in patients with hard mucin. This study aimed to assess whether combining collagenase or cysteamine with BromAc^**®**^ would be more effective in dissolving hard mucin.

**Methods:**

This preclinical human *in vitro* study examined the effect of adding collagenase to BromAc^**®**^ on hard mucin samples when incubated at 37 °C over 24 h. The effect of cysteamine alone and in combination with collagenase and BromAc^**®**^ was also examined as a supplementary arm of this study. Five experiments were conducted with appropriate controls. Human hard mucin samples were sliced into equal fractions using a sterile surgical scalpel, weighed and noted. The leftover solid mucin was weighed at 0, 1, 3, 5, and 24 h post-treatment.

**Results:**

At 24 h post-treatment, all combinations with collagenase demonstrated almost 100 % dissolution of hard mucin. At 3 and 5 h post-treatment, only BromAc^**®**^ with collagenase at 250 μg/mL was found to be superior to BromAc^**®**^ alone. Combinations with cysteamine were not found to be effective.

**Conclusions:**

This study provides promising evidence of the efficacy and synergistic effect of combining BromAc^**®**^ with collagenase to dissolve hard mucin. Further preclinical and clinical research should be undertaken to assess its safety and efficacy in the clinical setting.

## Introduction

Pseudomyxoma peritonei (PMP) is a rare peritoneal malignancy affecting approximately two in one million people, which typically originates from mucinous appendiceal tumours [1], 2]. It results in the progressive accumulation of mucinous material and tumour within the abdomen and pelvis [1], 2]. Untreated, the build-up of mucin leads to increasing abdominal distension as well as compression of the gastrointestinal tract, causing malnourishment and bowel obstructions [1], 2]. Mucin produced by PMP can be categorised as soft, semi-hard and hard [1]. This is based on various physical and chemical differences including the amount of cellular content, thiol distribution, hydration status and content of glucose, proteins and lipids [1]. Soft mucin is made up of 100 % glycoprotein, denser and has a higher concentration of sulfhydryl and disulphide bonds as well as higher amounts of protein, glucose and lipids [1]. Harder mucins have increasing amounts of cellular material and are accordingly less dense with lesser amounts of proteins, glucose and lipids and less sulfhydryl and disulphide bonds [1].

The current standard of curative treatment for PMP is cytoreductive surgery (CRS) with hyperthermic intraperitoneal chemotherapy (HIPEC); however, many patients require further operations with complication rates and technical difficulty increasing with each subsequent surgery [2], [3], [4]. For patients who are not operative candidates, limited treatment options exist as systemic chemotherapy is not typically effective in PMP [2], 3]. In this subset of patients, percutaneous drainage of the mucin to reduce the burden of disease can be considered [2].

BromAc^**®**^ is a novel mucolytic therapy developed to facilitate percutaneous drainage of mucin [1], 5], 6]. A combination of bromelain and N-acetylcysteine, BromAc^**®**^, breaks down the peptide and cysteine-disulphide bonds of the glycoprotein within mucin [1], 5], 6]. N-acetylcysteine further contributes to regenerating the active component of bromelain to potentiate its effects [6]. Preliminary trials have demonstrated BromAc^**®**^ to be a safe therapy; however, its effectiveness varies depending on the hardness of the mucin [1], 2], 5]. *In vitro* studies have demonstrated that while BromAc^**®**^ is 100 % effective in dissolving soft mucin, its effectiveness in semi-hard and hard mucin decreases to 60 and 40 %, respectively [1].

Thus, while BromAc^**®**^ is considered to be effective as a palliative treatment of PMP with soft mucin, there is a clinical need for the development of a combination therapy that is similarly effective in treating PMP that produces harder types of mucin.

A key reason for chemoresistance in harder tumours is due to the tumour stroma preventing the penetration of the drug [7]. The extracellular matrix of the tumour stroma is composed largely of collagen as well as cysteine-disulphide bonds, leading to the hypothesis that intraperitoneal administration of collagenase could therefore be effective in facilitating breakdown of the tumour stroma and enhanced penetration of intraperitoneal chemotherapy [7].

Collagenase is an enzyme that degrades collagen, a diverse family of proteins [8]. Collagen makes up about 30 % of the total protein content of the body and is the primary protein in the extracellular matrix [8]. All collagens have a common domain of three polypeptide chains [8]. Physiologically, collagenase promotes the degradation and re-synthesis of collagen fibres, which is essential for normal growth, wound healing and regeneration of tissue [8]. In the clinical setting, collagenase is a currently accepted treatment for the management of Dupuytren’s disease, Peyronie’s disease, debridement of cutaneous ulcers and burns management [8], [9], [10].

Cysteamine is an aminothiol and is a decarboxylated derivative of cysteine [11]. Cysteamine exerts a variety of actions under different conditions [11]. These include acting as an antioxidant, changing gene expression and changing enzymatic activity [11].

Given BromAc^**®**^ is 100 % effective in dissolving soft mucin that is composed solely of glycoprotein, it was postulated that the reduced effect of BromAc^**®**^ on hard mucin was due to its higher cellular content and hence more significant extracellular matrix. It was therefore hypothesised that the addition of collagenase to BromAc^®^ would facilitate degradation of this extracellular matrix and improve the penetration of BromAc^®^. Similarly, given the presence of cysteine-disulphide bonds within mucin, a secondary hypothesis was formed that the combination of bromelain with cysteamine with or without the addition of collagenase may also have an enhanced effect compared to BromAc^®^ alone [11].

## Materials and methods

The study was conducted with pre-existing approval from the St George Hospital Ethics Committee (approval number 2023/ETH01237). A frozen sample of hard mucin previously collected under sterile conditions and frozen immediately at a temperature of −80 °C was thawed in a 37 °C water bath on the day of each experiment. This mucin was collected intraoperatively from a human female patient with PMP. In this study, the same sample of mucin was used for each trial.

All chemical agents were procured from Sigma Aldrich Chemicals, Sydney, Australia. A standard commercially available Type IV collagenase (product code C5138) was used. Concentrations of bromelain, NAC and BromAc^**®**^ were chosen to reflect their current clinical use. Previous studies that have assessed the intraperitoneal use of collagenase have found a dose of 37.5 units/mL to be both safe and effective in both rat and pig models [7], 12]. This has accordingly informed the concentrations of collagenase tested in this study, which is approximately equivalent to 250 μg/mL of collagenase.

### Preparation of drug solutions

A total of five trials were conducted. All trials included 0.9 % saline, bromelain, NAC and collagenase as controls. The concentrations of controls were as follows:

– Bromelain (600 μg/mL),
– NAC (20 mg/mL),
– BromAc^**®**^ [Bromelain (600 μg/mL) with NAC (20 mg/mL)],
– Collagenase (62.5–500 μg/mL) (variation between trials).

Table 1 provides an overview of the solutions used for each trial. In summary, BromAc^**®**^ with varying concentrations of collagenase was assessed, as well as bromelain with collagenase and NAC with collagenase. Trials 1 and 2 additionally assessed the efficacy of combining bromelain with cysteamine as well as bromelain, cysteamine and collagenase. These two trials also had a cysteamine control included.

**Table 1:** Test solutions and controls across trials 1–5.

Trial	Controls^a^	Test solutions^a^
1	Saline Bromelain^b^ NAC^b^ Collagenase 500 Cysteamine 14 mg/mL BromAc^**®**b^	BromAc^**®**^ + cysteamine 14 mg/mL BromAc^**®**^ + collagenase 500 BromAc^**®**^ + collagenase 250 BromAc^**®**^ + collagenase 125
2	Saline Bromelain^b^ NAC^b^ Cysteamine 14 mg/mL Collagenase 500 BromAc^**®**b^	NAC 200 mg/mL Bromelain + cysteamine 14 mg/mL Bromelain + collagenase 500 NAC + collagenase 500 BromAc^**®**^ + collagenase 500 BromAc^**®**^ + collagenase 250 BromAc^**®**^ + collagenase 125 Bromelain + cysteamine 14 mg/mL + collagenase 500
3	Saline Bromelain^b^ NAC^b^ NAC 200 mg/mL Collagenase 250 BromAc^**®**b^	Bromelain + collagenase 250 NAC + collagenase 250 BromAc^**®**^ + collagenase 250 BromAc^**®**^ + collagenase 125 BromAc^**®**^ + collagenase 62.5 BromAc^**®**^ + collagenase 31.25
4	Saline Bromelain^b^ NAC^b^ Collagenase 250 BromAc^**®**b^	Bromelain + collagenase 250 NAC + collagenase 250 BromAc^**®**^ + collagenase 250 BromAc^**®**^ + collagenase 125 BromAc^**®**^ + collagenase 62.5 BromAc^**®**^ + collagenase 31.25
5	Saline Bromelain^b^ NAC^b^ BromAc^**®**b^ Collagenase 250 Collagenase 125 Collagenase 62.5	BromAc^**®**^ + collagenase 250 BromAc^**®**^ + collagenase 125 BromAc^**®**^ + collagenase 62.5 Bromelain + collagenase 250 Bromelain + collagenase 125 Bromelain + collagenase 62.5 NAC + collagenase 250 NAC + collagenase 125 NAC + collagenase 62.5

^a^Unless otherwise specified solutions in μg/ml. ^b^Concentrations of BromAc^**®**^, bromelain and NAC remained standard across all trials and solutions. BromAc^**®**^: bromelain 600 μg/ml + NAC 20 mg/ml, bromelain 600 μg/ml, NAC 20 mg/ml. NB NAC 200 mg/ml also used in trials 2 and 3 as specified above.

### Preparation and testing of hard mucin

(0.5 g) was measured for each sample and added to a test tube containing 5 mL of the test solution or control. The specimens were incubated in a 37 °C water bath for 24 h.

For the first trial, specimens were weighed at 0 and 24 h. For subsequent trials, specimens were weighed at 0, 1, 3, 5 and 24 h to provide further information on the activity of each solution over the 24-h period. Specimens were also photographed at each time point.

A digital analytical scale accurate to 0.001 g was used. A Petri dish was placed on the scale and the balance zeroed. For each sample of mucin at each time point, the mucin was transferred from the test tube using forceps to this Petri dish. Only mucin that was solid enough to be transferred with forceps was measured.

### Data analysis

Descriptive statistics including mean and standard deviation were performed for solutions that were tested over at least three of the five trials. Given the non-normal distribution of the data, the independent samples Kruskal–Wallis test and pairwise comparisons of solutions was used to determine statistical significance. Trial 1 was excluded from these calculations due to the missing data. The p-value was set at 0.05.

## Results

**Figure 1:**
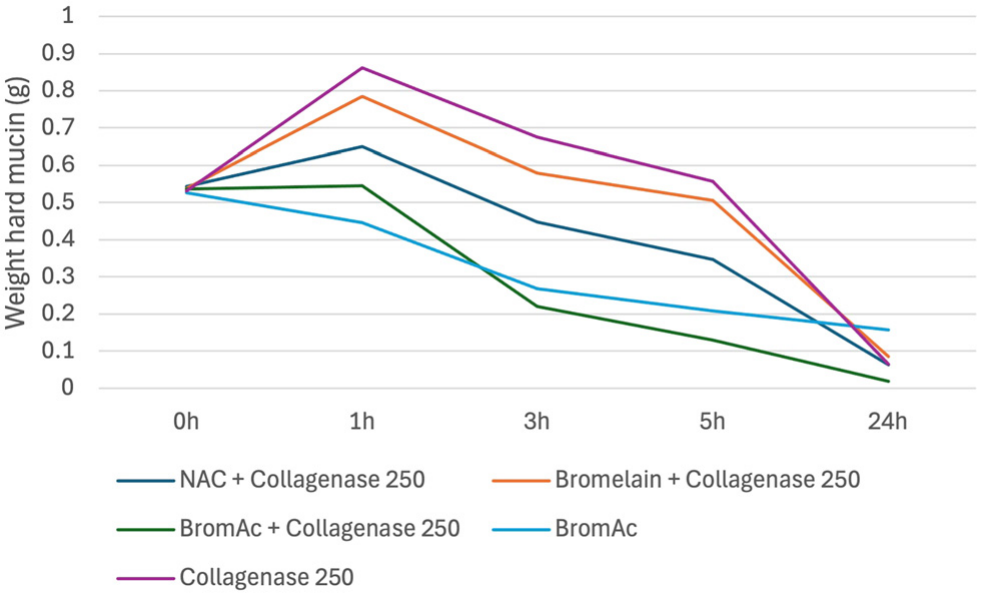
Average weight in hard mucin specimens over 0, 1, 3, 5 and 24 h when submersed in BromAc, BromAc + collagenase, NAC + collagenase, bromelain + collagenase and collagenase alone.

Overall, after 24-h of treatment, BromAc^**®**^, NAC and bromelain showed superior effects when combined with collagenase compared to BromAc^**®**^, NAC and bromelain alone. BromAc^**®**^, as well as the combination of BromAc^**®**^ with collagenase, demonstrated the fastest rates of degradation of hard mucin compared to all other combinations. BromAc^**®**^ with collagenase found to be most effective treatment as observed at 3- and 5-h post-treatment (Figure 1). These findings, however, were not statistically significant (Table 7, Appendix 2).

Appendix 1 outlines the results from all five trials, and Appendix 2 outlines the descriptive statistics carried out for the solutions that were tested in at least three trials. A wide range in standard deviation was noted across these data up to 57.2. The large standard deviation values suggest variability in the results and are likely due to the differing of solutions tested in each trial.

**Figure 2:**
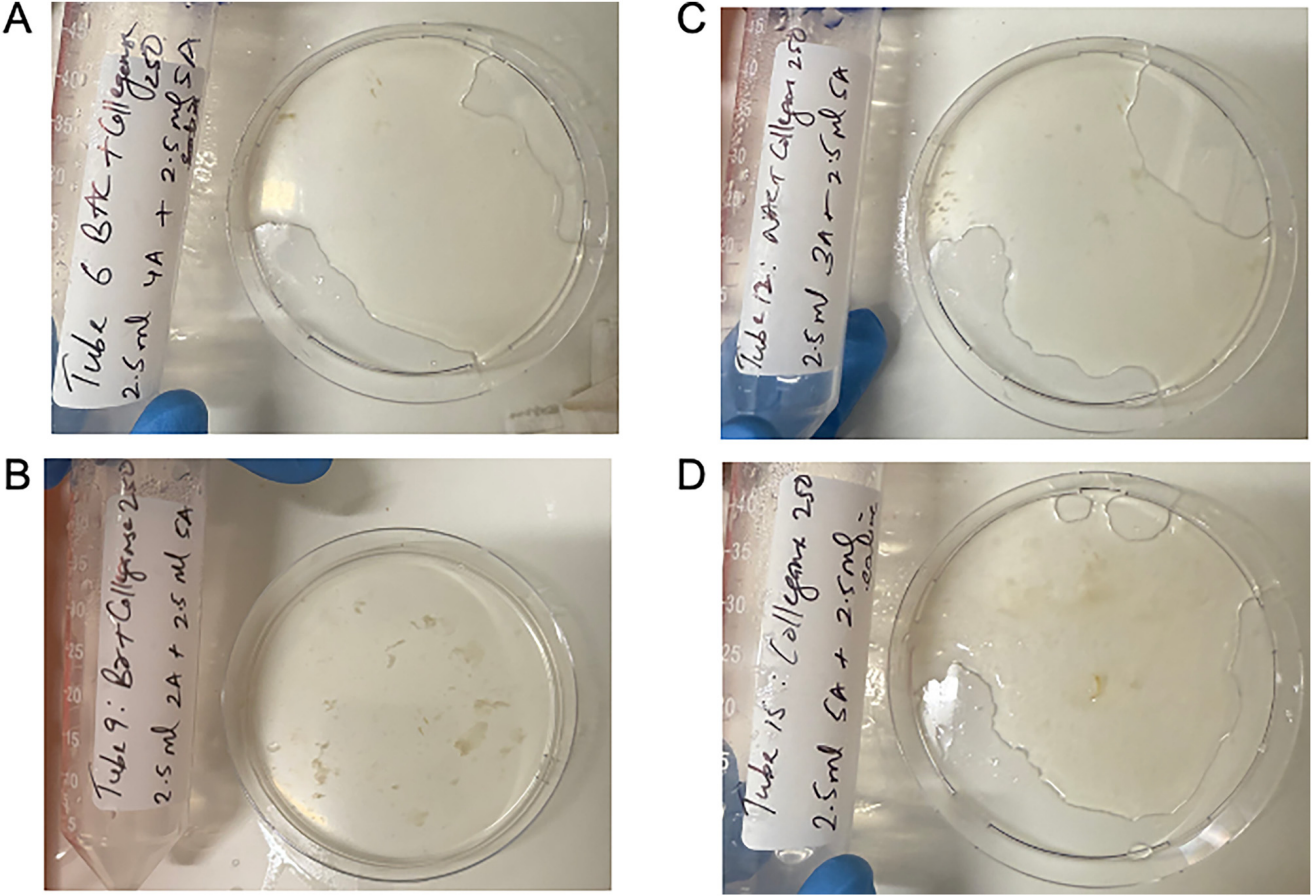
Quality of hard mucin dissolution in BromAc, bromelain and NAC with collagenase 250 μg/mL and collagenase 250 μg/mL control after 24 h. A: BromAc + collagenase 250. B: Bromelain + collagenase 250. C: NAC + collagenase 250. D: Collagenase 250.

The quality of dissolution also differed between solutions. Figure 2 demonstrates a sample of hard mucin that has been completely dissolved by BromAc^**®**^, NAC and bromelain in combination with collagenase, as well as collagenase alone, at 24 h. NAC with collagenase, bromelain with collagenase and collagenase alone all showed a progressively more gelatinous residual solution compared with the combination of BromAc^**®**^ with collagenase.

### Effect of BromAc^**®**^ with varying concentrations of collagenase

**Figure 3:**
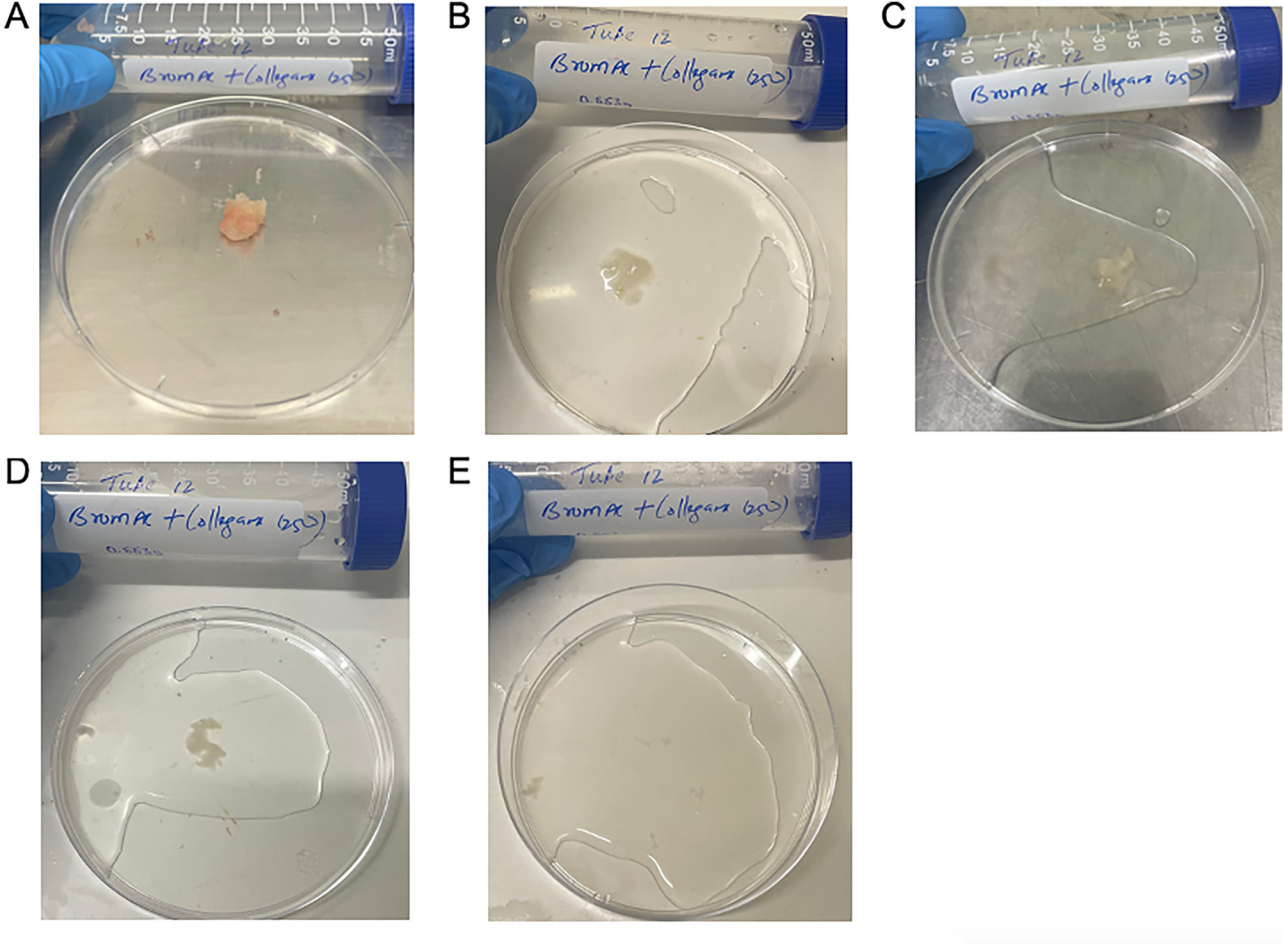
Qualitative demonstration of dissolution of hard mucin when submersed in BromAc^**®**^ + collagenase 250 ug/mL, trial 2. A: 0 h, B: 1 h, C: 3 h, D: 5 h, E: 24 h.

Figure 3 demonstrates a qualitative display of BromAc^**®**^ with collagenase at 250 μg/mL at each time point assessed.

**Figure 4:**
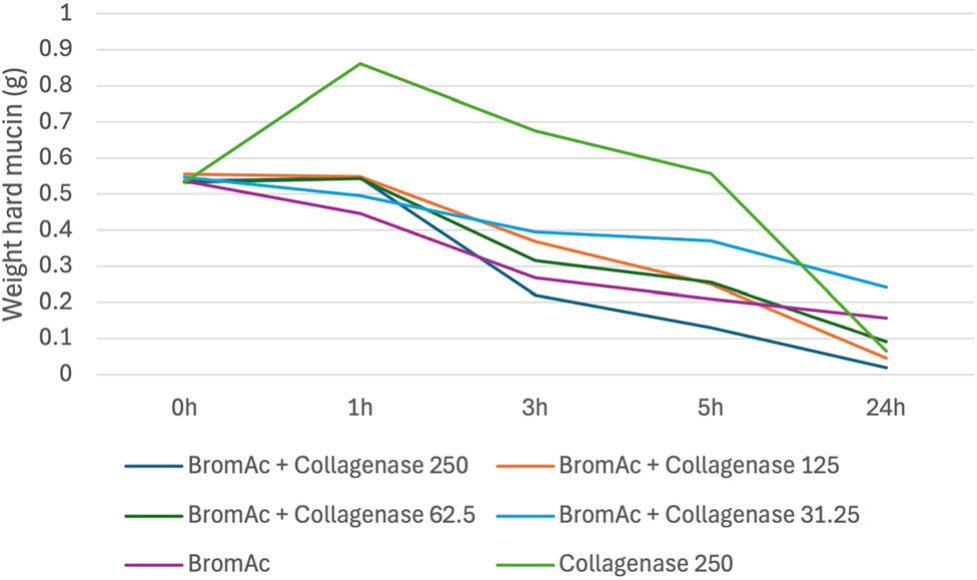
Average weight in hard mucin specimens over 0, 1, 3, 5 and 24 h when submersed in BromAc^**®**^ + collagenase at varying concentrations as well as BromAc^**®**^ and collagenase alone.

BromAc^**®**^ and collagenase demonstrated a dose–response relationship consistently across all trials with 100 % dissolution of the hard mucin after 24-h of treatment. Of all the solutions tested, only BromAc^**®**^ with collagenase consistently demonstrated a superior hard mucin dissolution effect compared to BromAc^**®**^ alone with an average of 96 % dissolution of hard mucin compared to 70 % at 24 h. Figure 4 outlines the average weights of hard mucin samples for BromAc^**®**^ with each concentration of collagenase tested at each time point. BromAc^**®**^ with collagenase at concentrations of 62.5, 125 and 250 μg/mL all demonstrated greater dissolution of mucin to BromAc^**®**^ alone at 24 h. However, when compared to BromAc^**®**^ alone, only BromAc^**®**^ and collagenase at 250 μg/mL consistently demonstrated superior effects at 3 and 5 h with an average of 59 % dissolution at 3 h and 76 % dissolution at 5 h compared to 50 % dissolution at 3 h and 61 % dissolution at 5 h for BromAc^**®**^ alone.

Collagenase alone demonstrated hydrational effect at 1 h post-treatment followed by a steady degradation rate to almost 100 % dissolution by 24 h of treatment.

### Additional solutions

#### The effect of NAC when combined with collagenase

**Figure 5:**
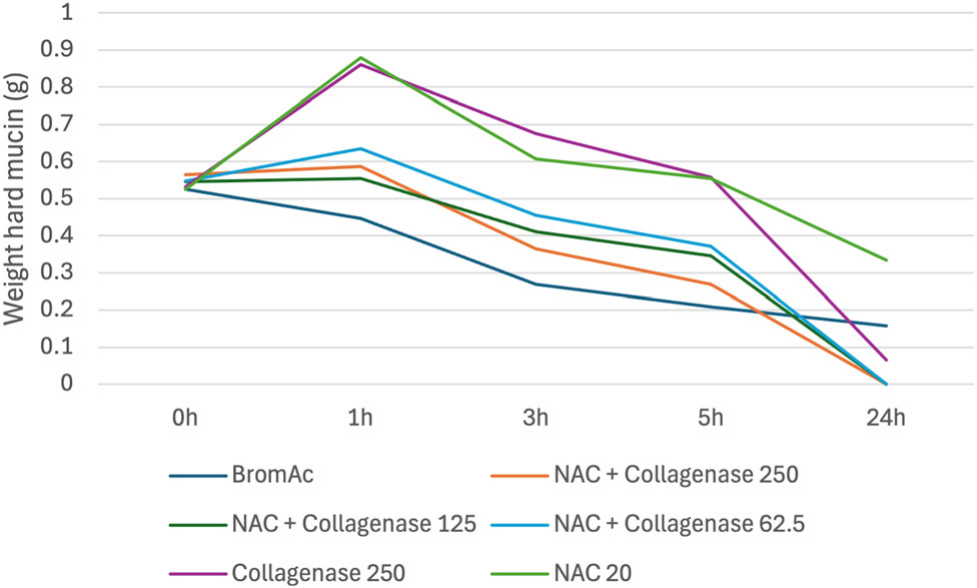
Average weight in hard mucin specimens over 0, 1, 3, 5 and 24 h when submersed in NAC + collagenase at varying concentrations as well as BromAc, NAC and collagenase alone.

While up to 100 % effective at 24 h, when compared to BromAc^**®**^ alone, NAC with collagenase was less effective at each earlier time point (Figure 5). The superior effect at 24 h was therefore attributed to the collagenase component.

#### The effect of bromelain when combined with collagenase

**Figure 6:**
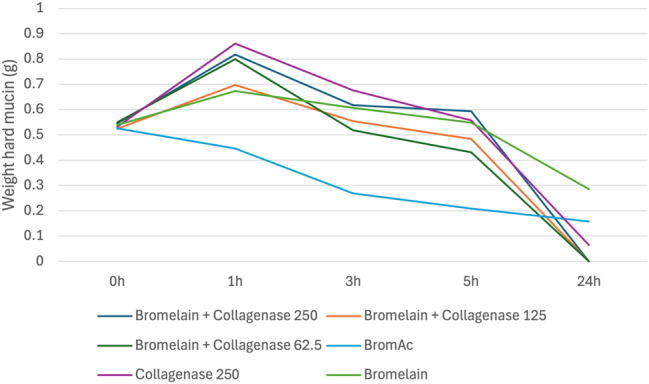
Average weight in hard mucin specimens over 0, 1, 3, 5 and 24 h when submersed in bromelain + collagenase at varying concentrations as well as BromAc, bromelain and collagenase alone.

Bromelain with collagenase was demonstrated to be less effective than BromAc^**®**^ alone and BromAc^**®**^ with collagenase. Its effect was similar to collagenase alone indicating that bromelain by itself is minimally effective in dissolving hard mucin [Figure 6].

#### The effect of bromelain/BromAc^**®**^ when combined with cysteamine

**Figure 7:**
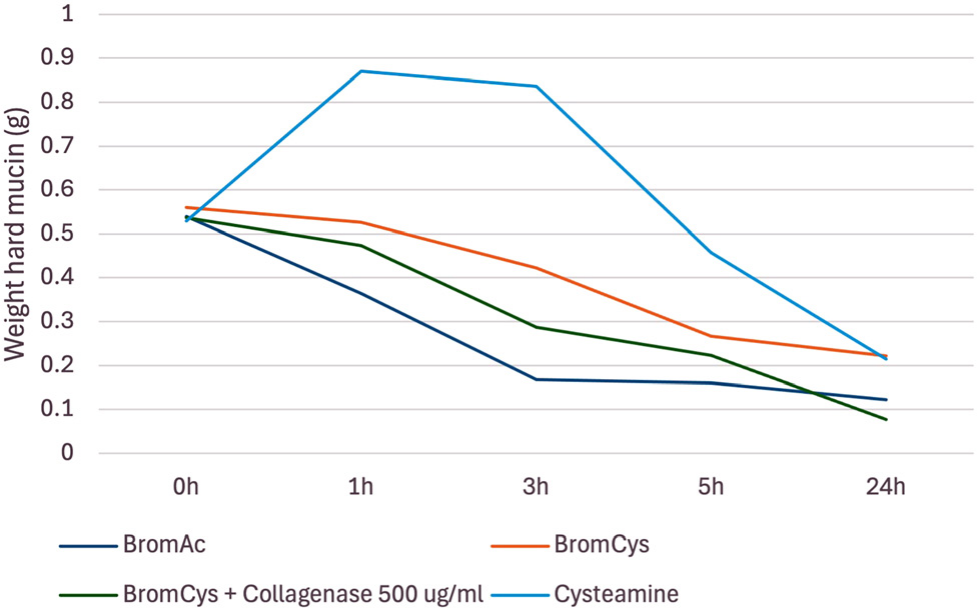
Average weight in hard mucin specimens over 0, 1, 3, 5 and 24 h when submersed in bromelain + cysteamine, bromelain + cysteamine + collagenase, and BromAc^**®**^ and cysteamine alone.

Bromelain with cysteamine demonstrated 52 % dissolution of hard mucin by 5 h with its effect plateauing to 60 % at 24 h. In trial 2, when bromelain and cysteamine were combined with collagenase, dissolution of hard mucin was enhanced up to 85 % at 24 h; however, it should be noted that this combination used a concentration of collagenase that was twice the current proven safe concentration. Given the combinations with cysteamine were overall found to be less effective than BromAc^**®**^ alone in the first two trials, it was hence excluded from ongoing trials [Figure 7].

## Discussion

In this study, BromAc^**®**^ with collagenase was the only combination that resulted in faster mucin degradation when compared to BromAc^**®**^ alone as well as all other tested solutions. The tumour stroma, composed largely of collagen, plays a key role in chemoresistance in hard tumours [7]. It is therefore hypothesised that intraperitoneal administration of collagenase facilitates break down of the tumour stroma, allowing enhanced penetration of other intraperitoneal therapies such as BromAc^®^.

Hard mucin treated with collagenase alone, as well as collagenase combined with bromelain, NAC and BromAc^®^ consistently demonstrated an increase in weight at 1 h followed by a linear decrease down to negligible quantities by 24 h. The increase in weight after 1 h was thought to be hydrational with the breakdown of the tumour stroma, resulting in the hard mucin absorbing water from the solution.

Given the abundance of collagen within the human body, there is a real safety concern regarding the effect of collagenase when administered into the abdomen, with complications such as catastrophic bleeding and gastrointestinal perforation known to occur in high doses [7], 12], 13]. Collagenase at a concentration of 250 μg/mL has already been demonstrated to be safe and effective in both rat and pig model studies. However, these studies have assessed its use over a relatively short timeframe [7], 12], 13].

BromAc^**®**^ is currently used in the non-operative setting with the dose administered via a percutaneous drain and left inside the abdomen for 24 h before aspirating and repeating the dose [5]. The question remains whether leaving BromAc^**®**^ and collagenase – in any concentration – is safe at 24 h or whether further studies should focus on BromAc^**®**^ and collagenase within a shorter timeframe, such as in the setting of HIPEC. A preclinical study has already demonstrated the addition of BromAc^**®**^ to HIPEC to be safe; thus, this line of research would also be feasible to pursue [14].

While BromAc^®^ with concentrations of 62.5–250 μg/mL of collagenase demonstrated superior results at 24 h compared to BromAc^®^ alone, only BromAc^®^ with collagenase 250 μg/mL demonstrated superior results at hours 3 and 5. This is clinically relevant as the synergistic effect of the two agents may shorten the exposure time of the proposed therapy and therefore limit potential toxic effects.

Hard mucin treated with cysteamine also demonstrated an increase in weight 1 h post-treatment which was presumed to be hydrational. By 24 h of treatment, the residual hard mucin quantities were very similar between cysteamine and bromelain with cysteamine. Bromelain with cysteamine was, however, consistently less effective than BromAc^®^ alone, suggesting less of a synergistic effect between bromelain and cysteamine compared with bromelain and NAC. Given the vastly inferior results with cysteamine in the first two trials, it was excluded from the remaining three trials to allow further concentrations of collagenase to be assessed.

This study was mostly limited by the relatively small number of trials as well as the variation in solutions tested in each trial. This reduced the reliability of the findings and hence compromised the ability to perform meaningful statistical analyses. While these findings did not demonstrate statistical significance, qualitatively there was a clear superior dissolution effect of BromAc^®^ with collagenase compared to BromAc^®^ alone. Alternative quantitative methods in addition to, or instead of, measuring weight difference alone should be considered for future studies. Rheology is the study of how materials deform and flow in response to applied forces [15]. Rheomuco is a rheometer designed specifically to examine the elastic, viscous and plastic properties of mucus [16]. Future studies could consider the use of Rheomuco to assess the properties of the hard mucin at each time point. While BromAc^®^ with collagenase may not result in 100 % dissolution of hard mucin, it may be that its effect reduces the mucin viscosity enough that it is able to be drained out.

This study also was carried out using hard mucin collected from a single patient. Future studies should include hard mucin collected from various patients to ensure that the findings are reliable and repeatable.

Another consideration is the effect that freezing the human hard mucin sample has had on its structural integrity. During the freezing process, ice forms in both intra and extracellular spaces, and it is understood that this damages the extracellular matrix structure; however, its subsequent impact on the post-thaw extracellular matrix structure integrity is incompletely understood [17]. Given it is hypothesised that the superior effect of BromAc^®^ with collagenase is secondary to the effect collagenase has on the extracellular matrix, it would be important to establish the exact effects of freezing and thawing mucin. Future studies should consider testing with fresh mucin samples or considering embedding mucin samples in cryoprotectant media before freezing, which has been demonstrated to preserve the mucin structure [18].

Furthermore, commercially, collagenase can be produced by various methods, most commonly by fermentation of *Clostridium histolyticum* due to the relative ease and lower cost of production [8]. There are six main types of collagenases, with each type having its maximum effect on different tissue types [8]. There is considerable variability in the types and combinations of collagenase used in current studies [7], 12], 13]. This study used type IV collagenase that was already available in the research lab. Whether a different type of collagenase or a combination of collagenases is most effective should be examined in future studies.

A final point of consideration is the pharmaceutical stability of BromAc^®^ with collagenase. This was not assessed during this study, however, would be relevant when considering its potential clinical uses and should be the focus of further studies.

## Conclusions

For patients with inoperable PMP, or who are otherwise not operative candidates, there is a current lack of effective treatments for the symptomatic management of hard mucin. This *in vitro* study using a human sample of hard mucin demonstrates the potential efficacy of combining collagenase with BromAc^**®**^ for this purpose; however, statistical analysis was limited due to the number and consistency of its trials. Future *in vitro* studies with a variety of hard mucin samples should be carried out to confirm efficacy, followed by *in vivo* studies with animal models to establish treatment safety.

## Supplementary Material

Supplementary Material

Supplementary Material
