# Supplementary material for: The addition of collagenase to BromAc ® for the management of inoperable pseudomyxoma peritonei – in vitro results
Source: Pleura Peritoneum. 2025 Oct 31;10(4):185–92. doi: 10.1515/pp-2025-0026 (PMC12707187; doi:10.1515/pp-2025-0026)
Supplement: Supplementary file 1 — Supplementary Material [file j_pp-2025-0026_suppl_001.docx]

# Appendix 1 – Results of each trial

## Solutions

All concentrations of collagenase are in mg/ml.

All bromelain solutions are in mg/ml and all NAC solutions in mg/ml.

BromAc was comprised of bromelain 600 mg/ml and NAC 20mg/ml.

BromCys was comprised of 600mg/ml bromelain and 14mg/ml cysteamine.

Cysteamine concentrations are in mg/ml.

## Trial 1

|  | Weight (g) | |
| --- | --- | --- |
| Solution | 0h | 24h |
| BromAc | 0.5 | 0.16 |
| BromAc + Collagenase 500 | 0.5 | 0 |
| BromAc + Collagenase 250 | 0.5 | 0.02 |
| BromAc + Collagenase 125 | 0.5 | 0.01 |

**Table 2** Trial 1 weight of hard mucin over selected time points.

## Trial 2

|  | Weight (g) | | | | |
| --- | --- | --- | --- | --- | --- |
| Solution | 0h | 1h | 3h | 5h | 24h |
| Saline | 0.519 | 0.733 | 0.795 | 0.863 | 0.881 |
| Bromelain 600 | 0.55 | 0.625 | 0.552 | 0.5 | 0.347 |
| NAC 20 | 0.522 | 0.797 | 0.49 | 0.469 | 0.274 |
| NAC 200 | 0.554 | 0.831 | 0.869 | 0.891 | 0.886 |
| Cysteamine 14 | 0.529 | 0.87 | 0.837 | 0.457 | 0.214 |
| Collagenase 500 | 0.577 | 1.003 | 0.794 | 0.641 | 0.121 |
| BromAc | 0.539 | 0.364 | 0.167 | 0.16 | 0.122 |
| BromCys | 0.56 | 0.526 | 0.423 | 0.267 | 0.221 |
| Bromelain 600ug/ml + Collagenase 500 | 0.515 | 0.754 | 0.501 | 0.324 | 0.078 |
| NAC 20mg/ml + Collagenase 500 | 0.53 | 0.483 | 0.283 | 0.178 | 0.027 |
| BromAc + Collagenase 500 | 0.55 | 0.533 | 0.283 | 0.2 | 0 |
| BromAc + Collagenase 250 | 0.553 | 0.328 | 0.171 | 0.043 | 0 |
| BromAc + Collagenase 125 | 0.556 | 0.596 | 0.407 | 0.308 | 0.044 |
| BromCys + Collagenase 500 | 0.536 | 0.474 | 0.287 | 0.223 | 0.077 |

**Table 3** Trial 2 weight of hard mucin over selected time points.

## Trial 3

|  | Weight (g) | | | | |
| --- | --- | --- | --- | --- | --- |
| Solution | 0h | 1h | 3h | 5h | 24h |
| Saline | 0.521 | 0.849 | 0.887 | 0.961 | 1.006 |
| Bromelain 600 | 0.501 | 0.562 | 0.477 | 0.39 | 0.193 |
| NAC 20 | 0.515 | 0.837 | 0.825 | 0.712 | 0.451 |
| NAC 200 | 0.502 | 0.713 | 0.733 | 0.694 | 0.76 |
| Collagenase 250 | 0.507 | 0.719 | 0.578 | 0.499 | 0.195 |
| BromAc | 0.515 | 0.466 | 0.3 | 0.241 | 0.18 |
| Bromelain 600 + Collagenase 250 | 0.539 | 0.821 | 0.672 | 0.606 | 0.257 |
| NAC 20 + Collagenase 250 | 0.546 | 0.777 | 0.673 | 0.52 | 0.189 |
| BromAc + Collagenase 250 | 0.533 | 0.605 | 0.418 | 0.306 | 0.073 |
| BromAc + Collagenase 125 | 0.547 | 0.597 | 0.489 | 0.336 | 0.066 |
| BromAc + Collagenase 62.5 | 0.519 | 0.624 | 0.456 | 0.332 | 0.146 |
| BromAc + Collagenase 31.25 | 0.525 | 0.65 | 0.559 | 0.515 | 0.307 |

**Table 4** Trial 3 weight of hard mucin over selected time points.

## Trial 4

|  | Weight (g) | | | | |
| --- | --- | --- | --- | --- | --- |
| Solution | 0h | 1h | 3h | 5h | 24h |
| Saline | 0.511 | 0.836 | 0.875 | 0.826 | 0.777 |
| Bromelain 600 | 0.553 | 0.774 | 0.694 | 0.656 | 0.214 |
| NAC | 0.523 | 1.038 | 0.508 | 0.565 | 0.303 |
| Collagenase 250 | 0.524 | 0.854 | 0.368 | 0.297 | 0 |
| BromAc | 0.558 | 0.463 | 0.261 | 0.188 | 0.133 |
| Bromelain 600ug + Collagenase 250 | 0.53 | 0.717 | 0.45 | 0.318 | 0 |
| NAC 20 + Collagenase 250 | 0.518 | 0.583 | 0.307 | 0.251 | 0 |
| BromAc + Collagenase 250 | 0.534 | 0.43 | 0.093 | 0.065 | 0 |
| BromAc + Collagenase 125 | 0.55 | 0.536 | 0.268 | 0.172 | 0 |
| BromAc + Collagenase 62.5 | 0.552 | 0.492 | 0.199 | 0.207 | 0.064 |
| BromAc + Collagenase 31.25 | 0.567 | 0.341 | 0.229 | 0.224 | 0.177 |

**Table 5** Trial 4 weight of hard mucin over selected time points.

## Trial 5

|  | Weight (g) | | | | |
| --- | --- | --- | --- | --- | --- |
| Solution | 0h | 1h | 3h | 5h | 24h |
| Saline | 0.533 | 0.874 | 0.85 | 0.843 | 0.87 |
| Bromelain 600 | 0.551 | 0.731 | 0.703 | 0.648 | 0.388 |
| NAC 20 | 0.54 | 0.842 | 0.609 | 0.473 | 0.31 |
| BromAc | 0.531 | 0.492 | 0.346 | 0.246 | 0.193 |
| BromAc + Collagenase 250 | 0.527 | 0.46 | 0.199 | 0.104 | 0 |
| BromAc + Collagenase 125 | 0.572 | 0.462 | 0.308 | 0.184 | 0.068 |
| BromAc + Collagenase 62.5 | 0.529 | 0.514 | 0.291 | 0.228 | 0.063 |
| Bromelain + Collagenase 250 | 0.545 | 0.818 | 0.617 | 0.594 | 0 |
| Bromelain + Collagenase 125 | 0.524 | 0.697 | 0.555 | 0.483 | 0 |
| Bromelain + Collagenase 62.5 | 0.55 | 0.8 | 0.518 | 0.431 | 0 |
| NAC + Collagenase 250 | 0.564 | 0.587 | 0.365 | 0.268 | 0 |
| NAC + Collagenase 125 | 0.546 | 0.554 | 0.41 | 0.345 | 0 |
| NAC + Collagenase 62.5 | 0.548 | 0.635 | 0.455 | 0.372 | 0 |
| Collagenase 250 | 0.565 | 1.01 | 1.082 | 0.877 | 0 |
| Collagenase 125 | 0.498 | 0.841 | 0.839 | 0.682 | 0.07 |
| Collagenase 62.5 | 0.524 | 0.803 | 0.932 | 0.743 | 0.122 |

**Table 6** Trial 5 weight of hard mucin over selected time points.
