# Supplementary material for: The addition of collagenase to BromAc ® for the management of inoperable pseudomyxoma peritonei – in vitro results
Source: Pleura Peritoneum. 2025 Oct 31;10(4):185–92. doi: 10.1515/pp-2025-0026 (PMC12707187; doi:10.1515/pp-2025-0026)
Supplement: Supplementary file 2 — Supplementary Material [file j_pp-2025-0026_suppl_002.docx]

# Appendix 2 –Statistical Findings

# Statistical Significance

|  | p-value | | | | |
| --- | --- | --- | --- | --- | --- |
| Trial | 0h | 1h | 3h | 5h | 24h |
| 2 | 0.448 | 0.448 | 0.448 | 0.448 | 0.448 |
| 3 | 0.443 | 0.443 | 0.443 | 0.443 | 0.443 |
| 4 | 0.440 | 0.440 | 0.440 | 0.440 | 0.440 |
| 5 | 0.451 | 0.451 | 0.451 | 0.451 | 0.451 |

**Table 7** Significance of weight distribution of hard mucin between each solution as determined by Independent-Samples Kruskal- Wallis Test where p-value is set at 0.05.

## Test Solutions

|  | N | Minimum | Maximum | Mean | Std. Deviation | Skewness | Std. Error |
| --- | --- | --- | --- | --- | --- | --- | --- |
| BromAc + Collagenase 250 | | | | | | | |
| 1h Percentage Change | 4 | -41 | 14 | -14.85 | 22.386 | .336 | 1.014 |
| 3h Percentage Change | 4 | -83 | -22 | -58.90 | 26.259 | 1.380 | 1.014 |
| 5h Percentage Change | 4 | -92 | -43 | -75.78 | 22.665 | 1.725 | 1.014 |
| 24h Percentage Change | 5 | -100 | -86 | -96.48 | 5.895 | 1.817 | .913 |
| BromAc + Collagenase 125 | | | | | | | |
| 1h Percentage Change | 4 | -19 | 7 | -1.88 | 12.502 | -1.295 | 1.014 |
| 3h Percentage Change | 4 | -51 | -27 | -37.78 | 12.843 | -.135 | 1.014 |
| 5h Percentage Change | 4 | -69 | -45 | -56.53 | 13.659 | -.004 | 1.014 |
| 24h Percentage Change | 5 | -100 | -88 | -94.08 | 4.817 | -.182 | .913 |
| NAC + Collagenase 250 | | | | | | | |
| 1h Percentage Change | 3 | 4 | 42 | 19.70 | 20.113 | 1.391 | 1.225 |
| 3h Percentage Change | 3 | -41 | 23 | -17.60 | 35.527 | 1.685 | 1.225 |
| 5h Percentage Change | 3 | -53 | -5 | -36.30 | 27.284 | 1.730 | 1.225 |
| 24h Percentage Change | 3 | -100 | -65 | -88.47 | 19.976 | 1.732 | 1.225 |
| Bromelain + Collagenase 250 | | | | | | | |
| 1h Percentage Change | 3 | 35 | 52 | 45.93 | 9.280 | -1.613 | 1.225 |
| 3h Percentage Change | 3 | -15 | 25 | 7.63 | 20.496 | -1.149 | 1.225 |
| 5h Percentage Change | 3 | -40 | 13 | -6.17 | 29.353 | -1.704 | 1.225 |
| 24h Percentage Change | 3 | -100 | -52 | -84.13 | 27.482 | 1.732 | 1.225 |

**Table 8** Descriptive statistics for test solutions.

## Controls

|  | N | Minimum | Maximum | Mean | Std. Deviation | Skewness | Std. Error |
| --- | --- | --- | --- | --- | --- | --- | --- |
| BromAc | | | | | | | |
| 1h Percentage Change | 4 | -32.50 | -7.40 | -16.6500 | 11.35327 | -1.294 | 1.014 |
| 3h Percentage Change | 4 | -69.10 | -34.90 | -49.7750 | 14.95268 | -.690 | 1.014 |
| 5h Percentage Change | 4 | -70.40 | -53.30 | -60.9500 | 8.75766 | -.176 | 1.014 |
| 24h Percentage Change | 5 | -77.40 | -63.70 | -70.0800 | 6.34169 | -.375 | .913 |
| NAC | | | | | | | |
| 1h Percentage Change | 4 | 53 | 99 | 67.45 | 21.105 | 1.781 | 1.014 |
| 3h Percentage Change | 4 | -6 | 60 | 15.98 | 30.626 | 1.598 | 1.014 |
| 5h Percentage Change | 4 | -13 | 38 | 5.93 | 23.469 | 1.212 | 1.014 |
| 24h Percentage Change | 5 | -48 | 88 | -11.36 | 57.245 | 1.941 | .913 |
| Collagenase 250 | | | | | | | |
| 1h Percentage Change | 3 | 4 | 42 | 19.70 | 20.113 | 1.391 | 1.225 |
| 3h Percentage Change | 3 | -41 | 23 | -17.60 | 35.527 | 1.685 | 1.225 |
| 5h Percentage Change | 3 | -53 | -5 | -36.30 | 27.284 | 1.730 | 1.225 |
| 24h Percentage Change | 3 | -100 | -65 | -88.47 | 19.976 | 1.732 | 1.225 |
| Bromelain | | | | | | | |
| 1h Percentage Change | 4 | 12 | 40 | 24.65 | 13.848 | .225 | 1.014 |
| 3h Percentage Change | 4 | -5 | 28 | 12.18 | 16.756 | -.069 | 1.014 |
| 5h Percentage Change | 4 | -22 | 19 | 1.27 | 20.266 | -.347 | 1.014 |
| 24h Percentage Change | 5 | -62 | 42 | -29.50 | 42.456 | 1.633 | .913 |

**Table 9** Descriptive statistics for control solutions.
